# Evaluating the Immunological Impact of Hepatitis B Vaccination in Patients with Inflammatory Bowel Disease

**DOI:** 10.3390/ijms27010531

**Published:** 2026-01-05

**Authors:** Irene Soleto, Alicia C. Marin, Montse Baldan-Martin, David Bernardo, María Chaparro, Javier P. Gisbert

**Affiliations:** 1 Gastroenterology Unit, Hospital Universitario de La Princesa, Instituto de Investigación Sanitaria Princesa (IIS-Princesa), Universidad Autónoma de Madrid (UAM), Centro de Investigación Biomédica en Red de Enfermedades Hepáticas y Digestivas (CIBEREHD), 28006 Madrid, Spain; 2 Viral Immunology Laboratory, Biomedicine Department, BICS Unit, Margarita Salas Centre for Biological Research (CIB-CSIC), 28040 Madrid, Spain; 3 Mucosal Immunology Lab, Instituto de Biología y Genética Molecular (IBGM, Universidad de Valladolid-CISC), 47003 Valladolid, Spain; 4 Centro de Investigación Biomédica en Red de Enfermedades Infecciosas (CIBERinfec), 28006 Madrid, Spain

**Keywords:** inflammatory bowel disease (IBD), hepatitis B virus (HBV), vaccination, regulatory T cell (Tregs)

## Abstract

Patients with inflammatory bowel disease (IBD) frequently fail to achieve protective immunity after hepatitis B vaccination, even with intensified vaccination schedules. In this observational real-world study, 18 patients with IBD who were seronegative for hepatitis B virus (HBV) received three standard doses of the Engerix-B^®^ vaccine (at 0, 1, and 6 months). After immunisation, patients were classified into responders and non-responders according to their serological response. Blood samples were collected before the first dose and after completion of the vaccination schedule. Responders activated pathways that supported durable protection, including conventional dendritic cells type 1 mobilisation, expansion of IgG plasmablasts, and preservation of B- and T-cell memory. In contrast, non-responders displayed a more inflammatory innate profile, characterised by enrichment of CCR2^+^ monocytes. They also showed higher baseline Treg frequencies, which may suppress effective effector responses, together with impaired natural killer (NK) activation and progressive loss of memory potential. This study shows that hepatitis B vaccine failure in inflammatory bowel disease reflects a convergence of excessive immune regulation, inflammatory activation, and loss of memory potential, underscoring that no single pathway can explain the impaired response.

## 1. Introduction

Hepatitis B virus (HBV) is a major etiologic factor for liver cirrhosis and hepatocellular carcinoma [1]. Since 1991, the World Health Organisation has recommended the inclusion of the HBV vaccination in routine childhood immunisation schedules [2,3]. The implementation of this strategy has significantly lowered the prevalence of the infection and the incidence of medical complications from chronic HBV infection. The prevalence of HBV infection in patients with inflammatory bowel disease (IBD) is similar to that in the general population. However, viral reactivation in immunocompromised individuals can lead to serious consequences [4]. Reactivation has been reported in up to one-third of IBD patients with positive HBV markers, causing severe complications, such as liver dysfunction, compared with an incidence of only 1% per year in the general population. All of these reactivations in patients affected by IBD have been described in individuals treated with immunosuppressive drugs (corticoids, thiopurines, methotrexate) or biologic drugs [5,6]. Given this, it is logical to consider the population of patients with IBD as a population of immunocompromised individuals with a high risk of getting HBV. Therefore, the European Crohn’s and Colitis Organisation (ECCO) recommends a systematic assessment of the immunological status of IBD patients regarding HBV and vaccination for all patients with negative serology at the time of IBD diagnosis [7].

In the general population, the response rate to HBV vaccination, defined as the development of antibody titres against the HBs antigen (anti-HBs) of ≥10 IU/L, is approximately 90% [8]. By contrast, our group and others have shown that patients with IBD respond in only 40–60% of cases [9,10,11,12]. In previous studies, patients treated with anti-TNF (Tumour Necrosis Factor antagonist) drugs showed significantly lower response rates compared with patients not receiving these therapies [13]. These observations highlight the need for optimised vaccination strategies to improve HBV protection in IBD. Engerix^®^ is a recombinant HBsAg vaccine formulated with aluminium hydroxide, whereas Fendrix^®^ incorporates the AS04 adjuvant (aluminium hydroxide plus monophosphoryl lipid A), designed to enhance early dendritic cell activation. Although AS04-containing vaccines generally induce stronger innate stimulation, clinical evidence in IBD patients has shown that a double-dose Engerix^®^ regimen is non-inferior to standard doses of Fendrix^®^ in patients with IBD in achieving seroconversion [12]. Several factors likely contribute to the reduced immunogenicity of the HBV vaccine in IBD patients. First, the vaccine-induced antibody production may be impaired in patients under immunosuppressive therapy [14,15]. Second, intrinsic immunological alterations associated with IBD pathogenesis may also blunt the responsiveness to antigens [16].

The success of the recombinant HBV vaccination depends mainly on the T-cell response against the vaccine antigen (Hepatitis B surface antigen, HBsAg). Antigen-presenting cells (mainly dendritic cells (DC)) must present this antigen to T cells, while proper T-B collaboration is required for B-cell proliferation and differentiation into plasma cells secreting anti-HBs antibodies [17]. Vaccine responsiveness is further influenced by host factors, including age, sex, genetic polymorphisms, comorbidities, immune status, and smoking [18]. Specific human leukocyte antigen (HLA) haplotypes have been consistently associated with impaired HBsAg recognition [19], representing the most well-defined genetic contributions to HBV vaccine hypo-responsiveness. Additional genetic variants outside the HLA region have also been proposed, although the evidence remains limited and less conclusive [20] Impaired CD4^+^ and CD8^+^ T-cell activity has been reported in non-responders [21,22,23], along with regulatory T-cell alterations in otherwise healthy individuals [24,25]. Similarly, diminished activation of NK and Natural Killer T cell (NKT) and their involvement in the response to the HBV vaccine were associated [26]. Possible genetic factors have also been described, although the data are still relatively scarce. On the other hand, the reduced effectiveness of the HBV vaccine has been described in conditions other than IBD that can affect the immune system. For example, it has been suggested that the HBV vaccine is less effective in patients with chronic viral infections (human immunodeficiency virus (HIV) and hepatitis C virus), due to a limited proliferative capacity of lymphocytes, attributed to changes introduced by the virus in the immune signalling mechanism [27]. An altered response of helper T cells has also been described in haemodialysis patients. In this regard, in some conditions, biological parameters predictive of the response to the HBV vaccine have been identified. For instance, a high count of helper T cells in patients with HIV or an elevated CD4/CD8 ratio in haemodialysis patients have been associated with a better vaccine response [28]. However, no cellular biomarkers are currently available to predict the HBV vaccine response in IBD. Moreover, the mechanisms underlying impaired vaccine responses in these patients remain poorly understood.

Considering that IBD is a multifactorial disease characterised by genetic variants affecting immune responses to environmental stimuli, including microbial recognition, it is critical to determine which immune cell populations contribute to HBV vaccine failure. This study aims to address this question by characterising the cellular mechanisms associated with the HBV vaccine response in IBD, identifying potential predictive factors, and ultimately guiding the development of more effective vaccination strategies for this vulnerable population.

## 2. Results

### 2.1. Cohort Characteristics and Between-Group Comparisons

A total of 18 IBD patients were included and classified according to their serological response to HBV vaccination with Engerix^®^, administered in three doses (baseline, one month, and six months). Responders (*n* = 8) were defined as those achieving anti-HBs antibody titres > 100 IU/L after completion of the full vaccination schedule, whereas non-responders (*n* = 10) had titres ≤ 100 UI/L. All patients were HBsAg-negative and anti-HBc-negative at baseline, thereby excluding previous natural HBV infection. Because all individuals were seronegative for HBV exposure markers, pre-vaccination anti-HBs testing was not clinically required; the anti-HBs concentrations shown in Table 1 correspond exclusively to post-vaccination titres.

The clinical and demographic characteristics are presented in Table 1. Among responders, three had Crohn’s disease (CD), and five had ulcerative colitis (UC), whereas among non-responders, five had CD and five had UC. The mean age was 58 years in both groups, and the female sex accounted for 50% of each group (4/8 responders, 5/10 non-responders). With respect to smoking status, non-responders comprised 4/10 current smokers (40%), 4/10 former smokers (40%), and 2/10 never smokers (20%), whereas responders comprised 1/8 current smokers (13%), 2/8 former smokers (25%), and 5/8 never smokers (63%).

Additional clinical variables, including BMI, comorbidities, diagnostic year, and IBD location, were comparable between the groups and did not differ significantly. Most patients were in clinical remission or exhibited only mild disease activity at the time of vaccination (Harvey–Bradshaw Index or partial Mayo score 0–1).

Altogether, none of the demographic or clinical variables analysed (age, sex, BMI, smoking status, disease type and location, disease duration, treatment, or comorbidities) differed significantly between responders and non-responders.

### 2.2. Dendritic Cell and Monocyte Subsets Do Not Differ Between Responders and Non-Responders at Baseline

**Figure 1:**
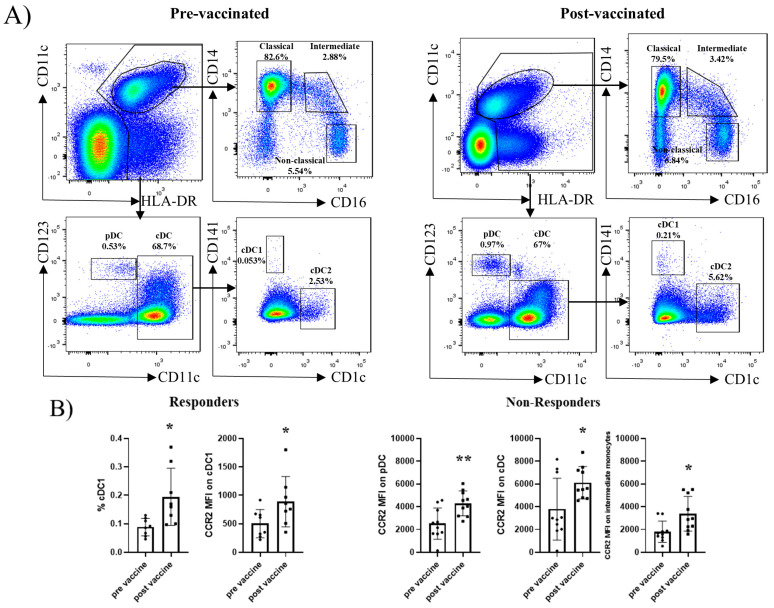
Dendritic cell (DC) and monocyte subsets in peripheral blood of patients with inflammatory bowel disease before and after hepatitis B vaccination. (**A**) Gating strategy shown for a representative patient before (pre-vaccinated) and after (post-vaccinated) HBV vaccination. Within singlet viable CD45^+^HLA-DR^+^ cells, plasmacytoid DC (pDC) were defined as CD123^+^CD1c^−^, while conventional DC (cDC) were identified as CD1c^+^ and further subdivided into cDC1 (CD141^+^) and cDC2 (CD1c^+^CD141^−^). From the same HLA-DR^+^ fraction, monocytes were gated as CD11c^+^ cells and classified into classical (CD14^+^CD16^−^), intermediate (CD14^+^CD16^+^), and non-classical (CD14^−^CD16^+^) subsets. (**B**) Quantification of DC and monocyte subsets, as well as CCR2 expression (MFI) within these populations, in responders and non-responders before and after HBV vaccination. Only those subsets and parameters showing statistically significant differences between pre- and post-vaccination are displayed. Data are presented as mean ± SEM. Statistical analysis was performed using a paired non-parametric Wilcoxon test. * *p* < 0.05, ** *p* < 0.01. Responders (*n* = 8), non-responders (*n* = 10).

Peripheral blood samples were collected from IBD patients before and after HBV vaccination, and DC and monocyte populations were defined as shown in Figure 1A. At baseline, the relative frequencies of plasmacytoid DC (pDC), conventional DC (cDC), and the different monocyte subsets did not differ significantly between responders and non-responders. Following vaccination, non-responders displayed a significant increase in CCR2 mean fluorescence intensity (MFI) within pDC, cDC, and transitional monocytes, whereas responders showed an increase in both the frequency of cDC1 and their CCR2 MFI (Figure 1B).

### 2.3. B-Cell Subsets Differ Between Responders and Non-Responders Before and After Hbv Vaccination

**Figure 2:**
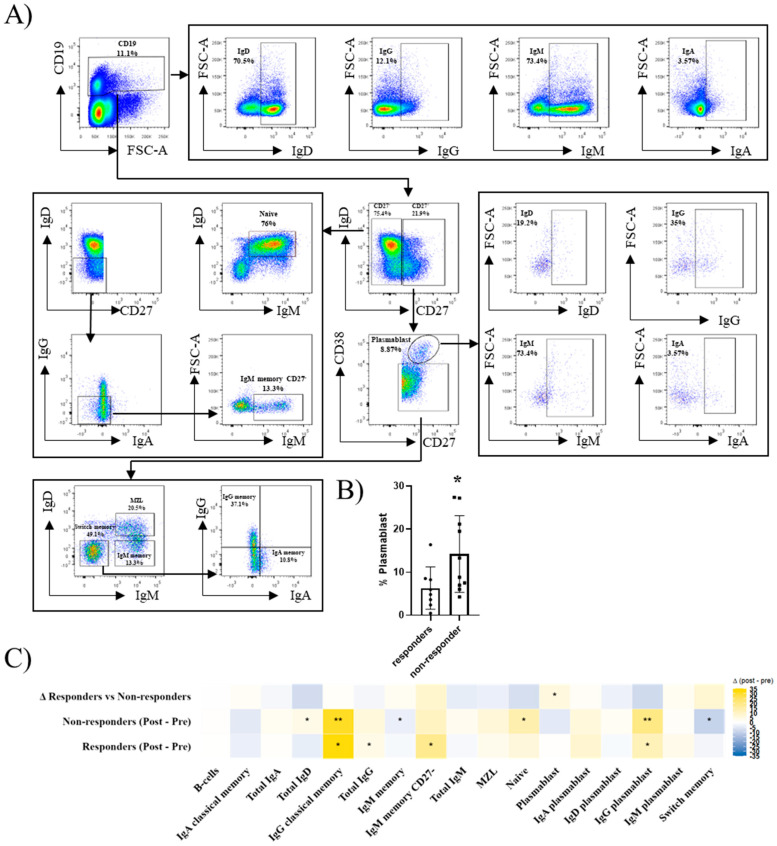
B-cell subset characterisation in IBD patients who respond or do not respond to HBV vaccination. (**A**) Gating strategy for the identification of B-cell subsets in peripheral blood. Viable CD45^+^CD19^+^ cells were selected, and IgD, IgM, IgG, and IgA expressions were analysed. CD19^+^ cells were subdivided based on CD27 expression. Within the CD27+ fraction, naïve B-cells were defined as IgD^+^IgM^+^. CD27^−^IgD^−^ cells were further analysed for IgG and IgA and IgM, with the last classified as IgM memory CD27^-^ cells. Within the CD27^+^ fraction, co-expression of CD38 and CD27 defined plasmablasts, which were further characterised for their expression of IgM, IgG, and IgA. CD27^+^CD38^-^ cells were subdivided according to the expression of IgD and IgM, with the double-positive cells classified as marginal zone-like B-cells, IgD^−^IgM^+^ cells as IgM memory B-cells, and IgD^-^IgM^−^ cells as class-switched memory B-cells. The latter were further divided into IgG memory (IgG^+^) and IgA memory (IgA^+^) subsets. (**B**) The graph shows the B-cell populations with differential expression between responder and non-responder groups at baseline. (**C**) The heatmap shows paired comparisons of B-cell subsets before and after vaccination. The lower row corresponds to responders, the middle row to non-responders, and the upper row to the comparison of deltas between both groups. Statistical analyses were performed using paired non-parametric Wilcoxon tests. * *p* < 0.05, ** *p* < 0.01. Responders (*n* = 8), non-responders (*n* = 10).

Peripheral B-cell subsets were also identified as shown in Figure 2A, both before and after HBV vaccination. At baseline, the non-responders exhibited a higher frequency of plasmablasts compared with responders (Figure 2B). Following vaccination, responders exhibited significant increases in IgG plasmablasts, IgM and IgG memory B-cells, as well as total IgG^+^ B-cells. In contrast, the non-responders displayed a reduction in memory B-cell subsets, including IgM memory and switched memory populations, accompanied by an increase in naïve (IgD^+^) B-cells. Non-responders also showed an increase in IgG classical memory B-cells and plasmablasts. Intergroup comparisons of vaccine-induced changes revealed that IgG plasmablasts increased in responders and decreased in non-responders, representing the most robust difference between the two groups (Figure 2C). Because the samples were obtained one month after the third vaccine dose, these findings reflect the post-contact remodelling of B-cell memory rather than the transient plasmablast peak observed shortly after antigen exposure.

### 2.4. T-Cell Subsets Differ Between Responders and Non-Responders Before and After Hbv Vaccination

**Figure 3:**
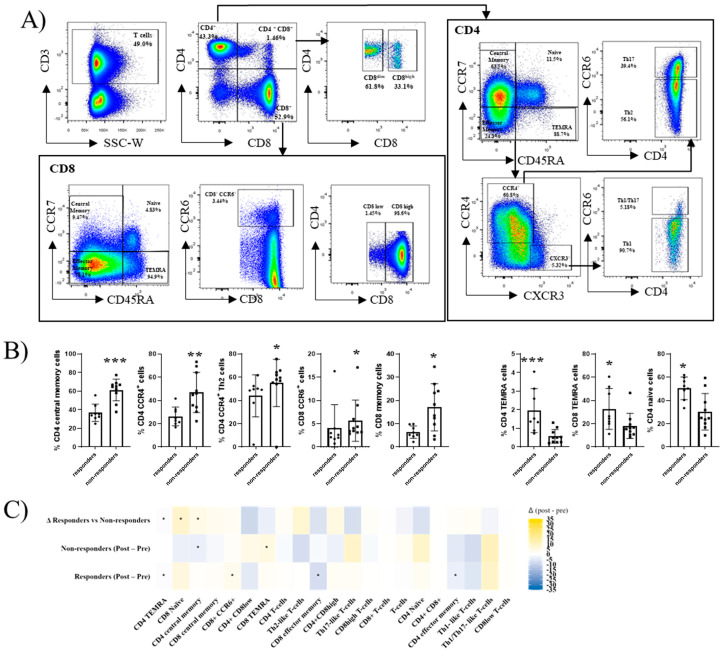
Peripheral T-cell immunophenotyping and response to HBV vaccination in IBD. (**A**) Gating strategy for T-cell subsets. Starting from singlet, viable CD45^+^ cells, and T cells were defined as CD3^+^. Within CD3^+^ cells, four fractions were identified according to CD4 and CD8 expression: CD4 T cells (CD4^+^CD8^−^), CD8 T cells (CD4^−^CD8^+^), double-positive (DP, CD4^+^CD8^+^), and double-negative (DN, CD4^−^CD8^−^). The DP and CD8 fractions were further split by CD8 expression level into CD8^dim and CD8^high. Within CD8 T cells, subsets were defined by CD45RA and CCR7 as Naïve (CD45RA^+^CCR7^+^), Central memory (CD45RA^−^CCR7^+^), and Effector memory (CD45RA^−^CCR7^−^). The TEMRA compartment here also comprised all CD8 T cells, excluding the Naïve subset. Expression of CCR6 was additionally assessed within CD8 T cells. Within CD4 T cells, the same scheme based on CD45RA/CCR7 defined Naïve, Central memory, Effector memory, and TEMRA subsets. Within CD4 TEMRA, cells were further divided by CCR4 and CXCR3 expression. CCR4^+^ TEMRA were subdivided into Th17-like (CCR6^+^) and Th2-like (CCR6^−^), while CXCR3^+^ TEMRA were subdivided into Th1/Th17-like (CCR6^+^) and Th1-like (CCR6^−^). (**B**) Baseline differences between groups. Bar plots display the baseline frequency (pre-vaccination) of the T-cell subset(s) differing between responders and non-responders. Dots represent individual patients; bars show mean ± SD. (**C**) Paired pre- vs. post-vaccination analysis summarised as a heatmap. Rows depict (from bottom to top): responders (post-vaccination–pre-vaccination), non-responders (post-vaccination–pre-vaccination), and the difference of deltas (Δ responders vs. non-responders). Each column is a T-cell subset. Colour encodes the signed median change after vaccination (yellow = increase; blue = decrease; white = no change). Asterisks inside tiles denote statistically significant results. All comparisons were performed with non-parametric Wilcoxon tests (paired when pre/post, unpaired between groups). *p* < 0.05 (*), *p* < 0.01 (**), *p* < 0.001 (***). Responders (*n* = 8), non-responders (*n* = 10).

Peripheral blood T-cell subsets were also analysed (Figure 3A). At baseline, non-responders exhibited significantly higher frequencies of CD4 memory T cells, CD4^+^CCR4^+^ (Th2-like) cells, CD8^+^CCR6^+^ cells, and CD8 memory T cells. In contrast, responders displayed higher proportions of CD4 naïve T cells and CD4/CD8 terminal effector memory T cells (TEMRA) subsets (Figure 3B).

Following vaccination, non-responders showed a decrease in CD4 memory T cells accompanied by an increase in CD8 TEMRA cells. In responders, vaccination induced a reduction in effector T cells (both CD4 and CD8), together with an increase in CD8^+^CCR6^+^ cells (Figure 3C). Direct comparison of the vaccine-induced changes between the groups revealed that the responders gained CD8 naïve and CD4 memory T cells, whereas the non-responders lost these subsets, representing a key divergence in T-cell dynamics after HBV vaccination. These patterns suggest that effective vaccine responses are linked to the maintenance of T-cell memory, whereas vaccine failure is associated with terminal differentiation.

### 2.5. Treg, Nk, and Nkt-Cell Subsets Differ Between Responders and Non-Responders After Hbv Vaccination

**Figure 4:**
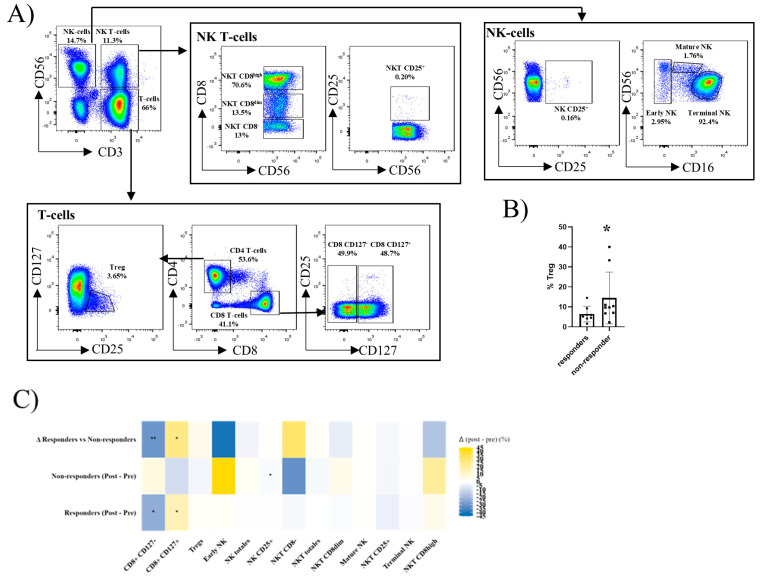
Peripheral Treg, NK, and NKT-cell immunophenotyping and response to HBV vaccination in IBD. (A) The gating strategy used to identify the main Treg, NK, and NKT subsets. Starting from singlet viable CD45^+^ leukocytes (negative for viability dye), T cells were defined as CD3^+^CD56^−^, NK cells as CD3^−^CD56^+^, and NKT cells as CD3^+^CD56^+^. Within T cells, CD8 T-cell (CD8^+^CD4^−^) and CD4 T-cell (CD4^+^CD8^−^) fractions were distinguished. CD8 T cells were further divided into two subgroups based on CD127 expression: CD8^+^CD127^+^ and CD8^+^CD127^−^. Within CD4 T cells, regulatory T cells (Treg) were defined as CD4^+^CD127^−^CD25^+^. NKT cells were subdivided according to CD8 expression into NKT CD8^high^, CD8^dim^, and CD8^-^, and the proportion of NKT CD25^+^ cells was also determined. NK cells were classified based on CD56 and CD16 expression into Early NK (CD56^+^CD16^−^), Mature NK (CD56^+^CD16^dim^), and Terminal NK (CD56+CD16+). The expression of CD25 within NK identified NK cells as CD25^+^. Arrows indicate the relationships across plots, and numbers above gates correspond to the identified subsets. (**B**) The graph shows the baseline comparison of significant subsets between responders and non-responders. Data are shown as mean ± SD; each dot represents an individual subject. (**C**) Paired pre- vs. post-vaccination analysis summarised as a heatmap. Rows depict (from bottom to top): responders (Post–Pre), non-responders (Post–Pre), and the difference of deltas (Δ responders vs. non-responders). Each column corresponds to a T-cell, NK T-cell and NK-cell subset. Colour encodes the signed median change after vaccination (yellow = increase; blue = decrease; white = no change). Asterisks inside tiles denote statistically significant results. All comparisons were performed with non-parametric Wilcoxon tests (paired for pre/post) or Mann–Whitney tests (between groups). *p* < 0.05 (*), *p* < 0.01 (**). Responders (*n* = 8), non-responders (*n* = 10).

Treg, NK, and NKT-cell populations were also analysed by flow cytometry, as shown in Figure 4A. At baseline, the non-responders exhibited significantly higher frequencies of regulatory T cells (Figure 4B).

In paired analyses, non-responders showed a significant decrease in the frequency of NK CD25-expressing NK cells after vaccination. In contrast, responders displayed remodelling of the CD8 compartment consistent with memory generation, characterised by a decrease in CD8^+^CD127^−^ cells and a reciprocal increase in CD8^+^CD127^+^ cells.

Direct comparison of the vaccine-induced changes confirmed these divergent patterns: responders reduced CD8^+^CD127^−^ and expanded CD8^+^CD127^+^ subsets, whereas non-responders showed the opposite response (Figure 4C). These findings suggest that elevated baseline regulation, impaired NK activation, and failure to consolidate CD8 memory are associated with vaccine non-response. Paired comparisons were assessed using Wilcoxon signed-rank tests, and between-group differences were evaluated with Mann–Whitney tests.

## 3. Discussion

This study provides an integrated view of innate and adaptive immune responses to HBV vaccination in patients with IBD. By analysing DC, monocytes, B cells, T cells, NK cells, NKT cells, and Tregs subsets before and after vaccination, we were able to visualise cellular programs that distinguished responders from non-responders. Our findings extend the previous knowledge by linking functional differences across multiple immune compartments with vaccine outcome in this clinical population, where impaired vaccine efficacy is well recognised. Importantly, these results should be interpreted in the context of the study’s overarching aim: to define the principal immunological differences that underlie failed vaccine responses in the setting of IBD-associated immune dysregulation.

In the innate compartment, the responders showed increased cDC1 and CCR2 expression, whereas the non-responders displayed an overall enrichment of CCR2^+^ cells among pDC, cDC, and transitional monocytes. This indicates that Engerix^®^ can activate myeloid cells, but the pattern of activation differs across compartments in each group. The responders mobilised cDC1, a subset strongly associated with effective antiviral T-cell priming [29]. In contrast, the predominance of CCR2^+^ inflammatory monocytes in non-responders is associated with immune profiles that have been linked to suboptimal antigen presentation and inflammatory trafficking in HBV-related contexts, rather than with efficient priming pathways [30]. These results are consistent with prior observations that HBV vaccines may induce relatively modest innate stimulation, and they align with clinical evidence indicating that IBD and deficits in dendritic cell activation may contribute to reduce vaccine responsiveness, an aspect that may help explain why optimised Engerix^®^ regimens can achieve seroconversion rates comparable to those of the AS04-adjuvant Fendrix^®^ [31,32].

Among the multiple immune compartments analysed, B-cell memory and plasmablast subsets represented the most statistically robust findings, with several differences remaining significant after FDR correction. In contrast, changes observed in innate, T-cell, and regulatory compartments should be interpreted as hypothesis-generating signals consistent with known immunological pathways. The responders had expanded IgG plasmablasts (1.8-fold, *p* = 0.012) and both IgM and IgG memory cells after vaccination (1.7-fold and 2.4-fold increases, *p* = 0.012 and *p* = 0.05, respectively), while the non-responders lost IgM memory subsets (0.67-fold, *p* = 0.022) and accumulated naïve cells (0.74-fold, *p* = 0.007). This pattern is in line with previous reports showing that protective vaccine responses depend on the generation of long-lived plasma cells and memory B-cells, whereas failure is associated with impaired memory formation [33]. Given that sampling occurred one month after the final vaccine dose, the increase in IgG plasmablasts observed in the responders likely represents the sustained remodelling of the antibody-secreting compartment, consistent with ongoing memory consolidation, rather than an early transient plasmablast peak. The divergence in IgG plasmablast trajectories was particularly robust (Δ median +1.6%, *p*= 0.03). Together, these data indicate that the quality of B-cell memory formation is a key candidate mechanism distinguishing a successful from a failed response in IBD [34,35].

T-cell analyses indicated that the responders preserved naïve and memory potential, whereas the non-responders showed an expansion of TEMRA cells. At baseline, the non-responders also displayed higher frequencies of CD4 memory and Th2-like CCR4^+^ subsets (1.7-fold and 1.3-fold increases, *p* = 0.001 and *p* = 0.016, respectively), while the responders had more CD4 naïve cells (1.7-fold higher, *p* = 0.013). This more differentiated and Th2-skewed baseline profile in non-responders suggests an immune environment less conducive to effective priming, whereas the naïve-biased repertoire in responders is compatible with higher plasticity and memory generation [36,37]. The reduction in effector CD4/CD8 cells (0.77-fold and 0.66-fold decreases, *p* = 0.04 and *p* = 0.03, respectively) and the increase in CD8 CCR6^+^ cells in responders (2.46-fold increases, *p* = 0.017) may reflect functional remodelling, whereas the loss of memory in non-responders suggests limited plasticity. Although functional confirmation is needed, the overlap with the prior literature supports the biological plausibility of these observations. Together, these features indicate that responders retain a T-cell landscape permissive to memory differentiation, whereas non-responders display baseline polarisation patterns commonly associated with suboptimal vaccine priming.

In the regulatory and NK compartments, the non-responders had higher baseline Tregs and showed a decline in NK CD25^+^ cells post-vaccination (fold change 0.03, *p* value = 0.038), while the responders remodelled CD8 subsets toward CD127^+^ memory-like cells, with a 1.41-fold increase (*p* = 0.011). This aligns with the concept that competition for IL-2 in the presence of abundant Tregs is associated with immune profiles compatible with increased regulatory pressure, which has been reported to limit effector expansion in other vaccination settings [38], while IL-7/CD127 signalling supports memory CD8 differentiation [39,40,41]. While Tregs have been proposed as candidate biomarkers of vaccine response [42], our study does not provide sufficient evidence to support this role. However, by integrating regulatory, innate, and CD8 dynamics, these findings suggest that differences in IL-2 and IL-7 associated signalling environments are linked to distinct patterns of vaccine responsiveness in IBD. We intercept CD25 on NK cells as a marker of IL-2 responsiveness rather than a standalone activation readout; definitive NK activation typically requires a complementary phenotypic or functional marker (e.g., CD69, HLA-DR, CD38, CD107a). The combination of elevated Treg frequencies and reduced NK IL-2 responsiveness in non-responders further supports the idea that early cytokine availability may be a limiting factor for downstream adaptive priming.

This work provides one of the first multi-compartmental analyses of HBV vaccine responses in IBD. The simultaneous profiling of innate, B, T, and regulatory cells before and after vaccination allows us to generate mechanistic hypotheses that go beyond serological endpoints.

Although exploratory in nature, the available sample size allowed the detection of large and biologically meaningful immunological differences between responders and non-responders. Some associations did not remain significant after FDR correction; however, their internal consistency and alignment with established immunological pathways suggest that they represent meaningful biological trends rather than random noise. Given the observational design and absence of functional assays, all mechanistic interpretations should be regarded as associative and require validation in future studies. Finally, although no external healthy or vaccine-naïve control group was included, the prospective design and internal comparison between responders and non-responders provide an internally consistent framework to uncover immune determinants of vaccine efficacy in this clinical population.

From a clinical standpoint, our study reinforces the importance of understanding the cellular mechanisms of vaccine efficacy in immunocompromised patients. Although we cannot propose Tregs or any single subset as a biomarker in this small cohort, the data add to current evidence that regulatory and innate balance influences HBV vaccine immunogenicity. By characterising a broad panel of subsets, our study extends the knowledge of how IBD patients respond to HBV vaccination and underscores the need for strategies that enhance memory formation and overcome regulatory dominance.

In conclusion, effective HBV vaccine responses in IBD were associated with the mobilisation of cDC1, expansion of plasmablasts and memory B-cells, preservation of T-cell memory potential, and remodelling of CD8 CD127^+^ subsets. Non-responders instead showed a more inflammatory innate profile, higher baseline Tregs, a reduced frequency of CD25-expressing NK cells, and the loss of memory potential. These findings collectively highlight that vaccine efficacy depends on the coordinated engagement of innate activation, balanced immune regulation, and adaptive memory preservation, rather than any single immune compartment, and they outline immunological axes that may be leveraged in future strategies.

## 4. Materials and Methods

### 4.1. Patients and Sample Collection

This was an observational real-world study aimed at evaluating factors associated with the immunogenicity of HBV vaccine in patients with IBD. Patients were recruited consecutively from the Inflammatory Bowel Disease Unit of Hospital Universitario de La Princesa (Madrid, Spain) during routine outpatient visits between 5 June 2018 and 18 November 2019, when hepatitis B vaccination was clinically indicated. Vaccination was prescribed as part of standard clinical care, and biological sample collection was performed prospectively within the vaccination schedule.

Biological samples were obtained from a total of 18 IBD patients included in the final analysis, 10 non-responders and 8 responders to vaccination (10 with UC and 8 with CD). In total, 28 patients initially met the inclusion criteria and were recruited. Of these, 10 patients were excluded from the final analysis after enrolment, in accordance with the predefined inclusion and exclusion criteria of the study, leaving 18 patients with complete pre- and post-data. The vaccination data are available for analysis.

The inclusion criteria comprised (1) a confirmed diagnosis of IBD and (2) first-time HBV vaccination as part of routine clinical care. The exclusion criteria included age < 18 years, positive HBV serology, advanced chronic disease or conditions preventing follow-up, immunodeficiency unrelated to IBD therapy, active infections, recent antibiotic use (within 30 days), allergy to vaccine components, pregnancy or lactation, substance abuse, or refusal to provide informed consent. Patients with active IBD were not excluded, reflecting the real-world nature of the cohort. Nevertheless, most participants were in clinical remission or had mild disease activity at the time of vaccination.

Responders were defined as those achieving anti-HBs titres ≥ 100 UI/L after completion of the vaccination schedule, whereas patients with titres < 100 IU/L were considered non-responders. The ≥100 IU/L threshold was selected to identify patients with a robust and durable response, in line with previous studies in immunocompromised populations, including IBD patients [9,12].

The patient demographic and clinical characteristics are summarised in Table 1. The study was approved by the local ethics committee at La Princesa Hospital (Madrid, Spain), and written informed consent was obtained from all participants.

From each patient, 20 mL of peripheral blood was collected before vaccination and again 1 month after completion of the vaccination schedule. Blood was drawn into lithium–heparin tubes, transported at room temperature, and processed within 2 h of collection to preserve cellular integrity. All participants received three standard doses of Engerix-B ^®^ vaccine (20 μg per dose, intramuscular, at 0, 1, and 6 months) according to clinical practice.

### 4.2. Blood Processing

Peripheral blood mononuclear cells (PBMC) were isolated by density-gradient centrifugation using Ficoll-Paque PLUS (Amersham Biosciences, Buckinghamshire, UK). The PBMC were washed twice with complete medium [RPMI 1640 (Sigma-Aldrich, Darmstadt, Germany), 100 μg/mL penicillin/streptomycin, 2 mM L-glutamine, 50 μg/mL gentamicin (Sigma-Aldrich), and 10% foetal bovine serum (TCS Cellworks, Northampton, UK)]. The PBMC were cryopreserved at −80 °C until use. Thawing was performed in a 37 °C water bath, followed by centrifugation at 1500 rpm for 5 min, and removal of the supernatant. After recovery, the PBMC were resuspended in PBS containing 1 mM EDTA and 0.02% sodium azide (Fluorescence-activated cell sorting, FACS buffer) and stained with fluorochrome-conjugated antibodies, as detailed below. All steps were conducted under standardised conditions to ensure reproducibility across samples.

### 4.3. Antibody Labelling

The PBMC were stained with a panel of monoclonal antibodies and analysed by flow cytometry. In all cases, a Live/Dead fixable near-IR dead cell stain kit (Molecular Probes, Eugene, OR, USA) was added to the cells before antibody staining, allowing the exclusion of dead cells from the analysis. Supplementary Table S1 shows the specificity, clone, fluorochrome, and sources of the antibodies used. Staining was carried out in FACS buffer on ice and in the dark for 20 min, after blocking nonspecific binding. T-cell, B-cell, and innate leukocyte subsets were identified within viable leukocytes and further assessed for maturation and differentiation maker expression.

The main panels and marker combinations used to define each immune subset were as follows:

T cells: CD3, CD4, CD8. CCR7, CD45RA, CCR6, CCR4, CXCR3;

B cells: CD19, IgD, IgM, IgG, IgA; CD27, CD38;

Monocytes and dendritic cells: CD11c, HLA-DR, CD14, CD16, CD123, CD141; CD1c;

Regulatory and NK cells: CD3, CD4, CD8, CD25, CD56; CD127, CD16.

### 4.4. Flow Cytometry and Data Analysis

Cells were acquired using a FACSCanto II (BD Biosciences), and the data were analysed with FlowJo (version 10.1). All analyses were restricted to the singlet viable fraction. Positive and negative gates were established using the fluorescence minus one (FMO) method.

Definition of immune subsets. All analyses were performed on singlet viable CD45^+^ leukocytes. T cells were defined as CD3^+^ CD56^-^, NK cells as CD3^−^ CD56^+^, and NKT cells as CD3^+^ CD56^+^. Within T cells, CD4 T cells were defined as CD4^+^CD8^−^ and CD8 T cells as CD4^−^ CD8^+^. Naïve and memory differentiation was defined using CCR7 and CD45RA, as follows: Naïve (CD45RA^−^CCR7^−^) and TEMRA (CD45RA^+^CCR7^−^). CCR6, CCR4, and CXCR3 expression were assessed and are indicated in Figure 3A to define CCR6^+^ and Th-like polarised TEMRA subsets. B cells were defined as CD19^+^ lymphocytes. Naïve B cells were defined as IgD^+^ IgM^+^, and memory B cells were defined based on CD27^+^ CD38^-^cells. IgM memory B cells were defined as IgD^−^IgM^+^, class-switched memory B cells as IgD^−^IgM^−^, and further divided into IgG^+^ (IgG memory) and IgA^+^ (IgA memory) populations. Plasmablasts were defined as CD27^+^CD38^+^ cells and were further classified by IgM/IgG/IgA expression.

For the innate compartment, analyses were performed within HLA-DR^+^ cells, as shown in Figure 1A. pDC were defined as CD123^+^CD1c^−^ and cDC as cDC1c^+^, with cDC1 defined as CD141^+^ and cDC2 as CD1c^+^ CD141^−^. Monocytes were gated as CD11c^+^ cells and classified as classical (CD14^+^ CD16^−^), intermediate (CD14^+^ CD16^+^), and non-classical (CD14^-^CD16^+^) subsets. Regulatory T cells were defined within CD3^+^CD56^−^CD4^+^cells as CD25^+^CD127^−^.

### 4.5. Statistical Analysis

All statistical analyses were performed using RStudio (version 2025.09.0). Depending on the design of each comparison, paired or unpaired non-parametric tests were applied (Wilcoxon signed-rank test for paired analyses and Mann–Whitney U test for unpaired comparisons). Statistical significance was defined as *p* < 0.05. The patient characteristics were summarised as the age, sex, and IBD treatment. Categorical variables were expressed as absolute numbers and percentages, and continuous variables were reported as the mean ± standard deviation (SD).

Given the cohort size (responders, *n* = 8; non-responders *n* = 10), the available sample size allowed detection of robust effects. Based on conventional power calculations (α = 0.05, 1−β = 0.8), the minimal detectable standardised effect size for between-group comparisons was approximately d~1.3, and for paired analysis, it was d_z_~0.6 [43]. These correspond to large effects, meaning that the sample size allows the detection of biologically relevant differences of large magnitude, even though smaller effects may remain undetected.

The full statistical results, including the raw *p*-values, FDR-adjusted *q*-values, and effect sizes for each immune subset, are provided in Supplementary Tables S2–S4.

## 5. Conclusions

This study identifies key immunological mechanisms underlying effective versus failed hepatitis B vaccine responses in patients with inflammatory bowel disease.

The responders showed higher frequencies of cDC1 and a coordinated expansion of B- and T-cell memory and compartments, consistent with the establishment of long-term protective immunity.

The non-responders exhibited a more regulatory baseline profile, with increased Treg burden, Th2 skewing, and a predominance of CCR2^+^ myeloid cells, together with a loss of effective memory after vaccination.

The differential remodelling of the CD8 compartment, along with the selective decrease in NK CD25^+^ cells in non-responders highlights specific immunological axes that determine the success or failure of vaccine responses.

Integrating these observations indicates that vaccine efficacy depends on the cooperation between appropriate innate activation, balanced immune regulation, and the preservation of adaptive memory, rather than on a single branch of the immune system.

From a clinical perspective, immune profiles characterised by high Treg burden and a polarisation toward Th2 responses, together with an increased presence of CCR2^+^ innate subsets, may help identify patients who could benefit from adjuvanted or reinforced vaccination strategies, an aspect that warrants confirmation in larger cohorts.

## Figures and Tables

**Table 1:** Clinical and demographic characteristics of IBD patients included in the study.

ID	Responder Status	Sex	Age (year)	Smoking	BMI	Comorbidities	IBD Type	Location	IHB or Partial mayo pre-Vaccination	Year of Diagnostic	Current Treatment	Anti-HBs titer (IU/L, Prost-Vaccination)
VAC01	non-responder	Male	70	Former	33.1	Hypothyroidism	Crohn’s disease	Ileal	0	2016	Levothyroxine	N/A
VAC02	non-responder	Male	60	Current	23.5	-	Ulcerative colitis	Extensive colitis	0	2005	Azathioprine	148
VAC03	non-responder	Female	55	Former	17.8	Hypothyroidism	Ulcerative colitis	Extensive colitis	0	2017	mesalazine, carbimazole	N/A
VAC04	non-responder	Male	73	Former	28.4	Benignprostatic hyperplasia	Crohn’s disease	Ileal	0	2017	Tamsulosine	N/A
VAC05	non-responder	Female	52	Current	24.5	-	Crohn’s disease	Ileocolic	0	2015	Azathioprine	N/A
VAC06	non-responder	Male	52	Never	20.3	-	Ulcerative colitis	Proctosigmoiditis	1	2013	Pentase	57
VAC07	non-responder	Male	41	Never	25.1	-	Ulcerative colitis	Extensive colitis	5	2018	Pentase	45
VAC08	non-responder	Female	50	Current	21	-	Crohn’s disease	Ileocolic	0	2019	-	67
VAC09	non-responder	Female	58	Current	25.5	-	Crohn’s disease	Ileocolic	0	2000	Azathioprine	N/A
VAC10	non-responder	Female	72	Former	29.4	Bronchial asthma	Ulcerative colitis	Left colitis	4	2019	Pentase	33
VAC11	responder	Female	45	Never	22.3	Rosacea	Ulcerative colitis	Extensive colitis	0	2014	Azathioprine	>1000
VAC12	responder	Female	70	Current	24.5	Hypercholesterolemia	Ulcerative colitis	Extensive colitis	0	2007	Pentase	>1000
VAC13	responder	Male	49	Never	23.5	-	Ulcerative colitis	Extensive colitis	0	2004	Mesalazine	>1000
VAC14	responder	Male	49	Never	24.3	-	Ulcerative colitis	Left colitis	0	1995	Pentase	>1000
VAC15	responder	Female	62	Former	16.3	-	Ulcerative colitis	Extensive colitis	1	2018	Mesalazine	>1000
VAC16	responder	Male	62	Never	22.4		Crohn’s disease	Ileocolic	0	2000	Azathioprine	389.34
VAC17	responder	Female	60	Never	21.5	-	Crohn’s disease	Ileal	0	2018	Azathioprine	>1000
VAC18	responder	Male	65	Former	33.9	Diabetes mellitus, hepatic steatosis	Crohn’s disease	Ileal	0	2018	Metformin	>1000

## Data Availability

The original contributions presented in this study are included in the article/Supplementary Materials. Further inquiries can be directed to the corresponding authors.

## Supplementary Materials

The following supporting information can be downloaded at https://www.mdpi.com/article/10.3390/ijms27010531/s1.

## Abbreviations

The following abbreviations are used in this manuscript:

cDC	Conventional DC
CD	Crohn’s disease
DC	Dendritic cell
FACs	Fluorescence-activated cell sorting
FMO	Fluorescence minus one
HBV	Hepatitis B virus
HBsAg	Hepatitis B surface antigen
HIV	Human immunodeficiency virus
HLA	Human leukocyte antigen
IBD	Inflammatory bowel disease
MFI	Mean fluorescence intensity
NK	Natural killer
NKT	Natural killer T cell
PBMC	Peripheral blood mononuclear cells
pDC	Plasmacytoid DC
TEMRA	Terminal effector memory T cells
TNF	Tumour necrosis factor
Tregs	Regulatory T cell
UC	Ulcerative colitis

## References

[1] D’souza S., Lau K.C., Coffin C.S., Patel T.R. (2020). Molecular mechanisms of viral hepatitis induced hepatocellular carcinoma. World J. Gastroenterol..

[2] Belle Jarvis S., Fenton-Lee T., Small S. (2023). Introduction of the Hepatitis B Vaccine—Birth Dose: Methods of Improving Rates in a Milieu of Vaccine Hesitancy. Vaccines.

[3] Lavanchy D. (2004). Hepatitis B virus epidemiology, disease burden, treatment, and current and emerging prevention and control measures. J. Viral Hepat..

[4] Gisbert J.P., Chaparro M., Esteve M. (2011). Review article: Prevention and management of hepatitis B and C infection in patients with inflammatory bowel disease. Aliment. Pharmacol. Ther..

[5] Esteve M. (2004). Chronic hepatitis B reactivation following infliximab therapy in Crohn’s disease patients: Need for primary prophylaxis. Gut.

[6] Raimondo G., Navarra G., Mondello S., Costantino L., Colloredo G., Cucinotta E., Di Vita G., Scisca C., Squadrito G., Pollicino T. (2008). Occult hepatitis B virus in liver tissue of individuals without hepatic disease. J. Hepatol..

[7] Kucharzik T., Ellul P., Greuter T., Rahier J.F., Verstockt B., Abreu C., Albuquerque A., Allocca M., Esteve M., Farraye F.A. (2021). ECCO Guidelines on the Prevention, Diagnosis, and Management of Infections in Inflammatory Bowel Disease. J. Crohns Colitis.

[8] Di Lello F.A., Martínez A.P., Flichman D.M. (2022). Insights into induction of the immune response by the hepatitis B vaccine. World J. Gastroenterol..

[9] Singh A.K., Jena A., Mahajan G., Mohindra R., Suri V., Sharma V. (2022). Meta-analysis: Hepatitis B vaccination in inflammatory bowel disease. Aliment. Pharmacol. Ther..

[10] Ulrich J.A., Habash N.W., Ismail Y.A., Tremaine W.J., Weaver A.L., Murray J.A., Loftus E.V., Absah I. (2023). Effectiveness of Hepatitis B Vaccination for Patients with Inflammatory Bowel and Celiac Disease. Clin. Gastroenterol. Hepatol..

[11] Gisbert J.P., Menchén L., García-Sánchez V., Marín I., Villagrasa J.R., Chaparro M. (2012). Comparison of the effectiveness of two protocols for vaccination (standard and double dosage) against hepatitis B virus in patients with inflammatory bowel disease. Aliment. Pharmacol. Ther..

[12] Chaparro M., Gordillo J., Domènech E., Esteve M., Acosta M.B.-D., Villoria A., Iglesias-Flores E., Blasi M., Naves J.E., Benítez O. (2020). Fendrix vs Engerix-B for Primo-Vaccination Against Hepatitis B Infection in Patients with Inflammatory Bowwithisease: A Randomized Clinical Trial. Am. J. Gastroenterol..

[13] Gisbert J.P., Villagrasa J.R., Rodríguez-Nogueiras A., Chaparro M. (2012). Efficacy of Hepatitis B Vaccination and Revaccination and Factors Impacting on Response in Patients with Inflammatory Bowel Disease. Am. J. Gastroenterol..

[14] Andrade P., Santos-Antunes J., Rodrigues S., Lopes S., Macedo G. (2015). Treatment with infliximab or azathioprine negatively impact the efficacy of hepatitis B vaccine in inflammatory bowel disease patients. J. Gastroenterol. Hepatol..

[15] Cao Y., Zhao D., Xu A.-T., Shen J., Ran Z.-H. (2015). Effects of Immunosuppressants on Immune Response to Vaccine in Inflammatory Bowel Disease. Chin. Med. J..

[16] Cekic C., Aslan F., Kirci A., Gümüs Z.Z., Arabul M., Yüksel E.S., Vatansever S., Yurtsever S.G., Alper E., Ünsal B. (2015). Evaluation of Factors Associated with Response to Hepatitis B Vaccination in Patients with Inflammatory Bowel Disease. Medicine.

[17] Marín A.C. (2015). Immunogenicity and mechanisms impairing the response to vaccines in inflammatory bowel disease. World J. Gastroenterol..

[18] Blaine Hollinger F. (1989). Factors influencing the immune response to hepatitis B vaccine, booster dose guidelines, and vaccine protocol recommendations. Am. J. Med..

[19] Akcay I.M., Katrinli S., Ozdil K., Doganay G.D., Doganay L. (2018). Host genetic factors affecting hepatitis B infection outcomes: Insights from genome-wide association studies. World J. Gastroenterol..

[20] Xie B., Zhang P., Liu M., Zeng W., Yang J., Liu H. (2016). Deltex1 Polymorphisms Are Associated with Hepatitis B Vaccination Non-Response in Southwest China. PLoS ONE.

[21] Chedid M.G., Deulofeut H., Yunis D.E., Lara-Marquez M.L., Salazar M., Deulofeut R., Awdeh Z., Alper C.A., Yunis E.J. (1997). Defect in Th1-Like Cells of Nonresponders to Hepatitis B Vaccine. Hum. Immunol..

[22] Shokrgozar M.A., Shokri F. (2001). Enumeration of hepatitis B surface antigen-specific B lymphocytes in responder and non-responder normal individuals vaccinated with recombinant hepatitis B surface antigen. Immunology.

[23] Weihrauch M.R., von Bergwelt-Baildon M., Kandic M., Weskott M., Klamp W., Rösler J., Schultze J.L. (2008). T cell responses to hepatitis B surface antigen are detectable in non-vaccinated individuals. World J. Gastroenterol..

[24] He S., Li M., Ma X., Lin J., Li D. (2010). CD4^+^ CD25^+^ Foxp3^+^ Regulatory T Cells Protect the Proinflammatory Activation of Human Umbilical Vein Endothelial Cells. Arterioscler. Thromb. Vasc. Biol..

[25] Suzuki T., Yamauchi K., Kuwata T., Hayashi N. (2001). Characterization of hepatitis B virus surface antigen-specific CD4+ T cells in hepatitis B vaccine non-responders. J. Gastroenterol. Hepatol..

[26] Fisicaro P., Rossi M., Vecchi A., Acerbi G., Barili V., Laccabue D., Montali I., Zecca A., Penna A., Missale G. (2019). The Good and the Bad of Natural Killer Cells in Virus Control: Perspective for Anti-HBV Therapy. Int. J. Mol. Sci..

[27] Yao Z.Q., Moorman J.P. (2013). Immune Exhaustion and Immune Senescence: Two Distinct Pathways for HBV Vaccine Failure During HCV and/or HIV Infection. Arch. Immunol. Ther. Exp..

[28] Sari F., Taskapan H. (2012). Good response to HBsAg vaccine in dialysis patients is associated with high CD4+/CD8+ ratio. Int. Urol. Nephrol..

[29] Li X., Sun W., Xu X., Jiang Q., Shi Y., Zhang H., Yu W., Shi B., Wan S., Liu J. (2025). Hepatitis B virus surface antigen drives T cell immunity through non-canonical antigen presentation in mice. Nat. Commun..

[30] She S., Ren L., Chen P., Wang M., Chen D., Wang Y., Chen H. (2022). Functional Roles of Chemokine Receptor CCR2 and Its Ligands in Liver Disease. Front. Immunol..

[31] Hu M., Su Z., Yin Y., Li J., Wei Q. (2012). Calcineurin B subunit triggers innate immunity and acts as a novel Engerix-B^®^ HBV vaccine adjuvant. Vaccine.

[32] Lee G.-H., Lim S.-G. (2021). CpG-Adjuvanted Hepatitis B Vaccine (HEPLISAV-B^®^) Update. Expert Rev. Vaccines.

[33] Zhu D., Liu L., Yang D., Fu S., Bian Y., Sun Z., He J., Su L., Zhang L., Peng H. (2016). Clearing Persistent Extracellular Antigen of Hepatitis B Virus: An Immunomodulatory Strategy To Reverse Tolerance for an Effective Therapeutic Vaccination. J. Immunol..

[34] Schnell A., Schwarz B., Wahlbuhl M., Allabauer I., Hess M., Weber S., Werner F., Schmidt H., Rechenauer T., Siebenlist G. (2021). Distribution and Cytokine Profile of Peripheral B Cell Subsets Is Perturbed in Pediatric IBD and Partially Restored During a Successful IFX Therapy. Inflamm. Bowel Dis..

[35] Castro-Dopico T., Colombel J.-F., Mehandru S. (2020). Targeting B cells for inflammatory bowel disease treatment: Back to the future. Curr. Opin. Pharmacol..

[36] Sabry R., Mohamed Z.A.Z., Abdallah A.M. (2018). Relationship between Th1 and Th2 cytokine serum levels and immune response to Hepatitis B vaccination among Egyptian health care workers. J. Immunoassay Immunochem..

[37] Su J., Brunner L., Oz E.A., Sacherl J., Frank G., Kerth H.A., Thiele F., Wiegand M., Mogler C., Aguilar J.C. (2023). Activation of CD4 T cells during prime immunization determines the success of a therapeutic hepatitis B vaccine in HBV-carrier mouse models. J. Hepatol..

[38] Pallmer K., Oxenius A. (2016). Recognition and Regulation of T Cells by NK Cells. Front. Immunol..

[39] Bachmann M.F., Wolint P., Schwarz K., Jäger P., Oxenius A. (2005). Functional Properties and Lineage Relationship of CD8+ T Cell Subsets Identified by Expression of IL-7 Receptor α and CD62L. J. Immunol..

[40] Huster K.M., Busch V., Schiemann M., Linkemann K., Kerksiek K.M., Wagner H., Busch D.H. (2004). Selective expression of IL-7 receptor on memory T cells identifies early CD40L-dependent generation of distinct CD8^+^ memory T cell subsets. Proc. Natl. Acad. Sci. USA.

[41] Bachmann M.F., Beerli R.R., Agnellini P., Wolint P., Schwarz K., Oxenius A. (2006). Long-lived memory CD8^+^ T cells are programmed by prolonged antigen exposure and low levels of cellular activation. Eur. J. Immunol..

[42] Boer M.C., Joosten S.A., Ottenhoff T.H.M. (2015). Regulatory T-Cells at the Interface between Human Host and Pathogens in Infectious Diseases and Vaccination. Front. Immunol..

[43] Cohen J. (2013). Statistical Power Analysis for the Behavioral Sciences.

