# Supplementary material for: Evaluating the Immunological Impact of Hepatitis B Vaccination in Patients with Inflammatory Bowel Disease
Source: Int J Mol Sci. 2026 Jan 5;27(1):531. doi: 10.3390/ijms27010531 (PMC12787125; doi:10.3390/ijms27010531)
Supplement: Supplementary file 1 [file ijms-27-00531-s001.zip › ijms-4069224-supplementary.pdf]

Marker	Fluorochrome	Clone	Manufacturer
CD11c	FITC	HL3	Becton Dickinson
CCR2	PE	K036C2	Biolegend
CD14	PerCP-Cy5.5	M5E2	Biolegend
CD1c	PE-Cy7	L161	Biolegend
CD123	APC	6H6	Biolegend
CD16	APC-Cy7	3G8	Becton Dickinson
CD141	BV421	AD5-14H12	Miltenyi Biotec
HLA-DR	BV510	L243	Biolegend
IgD	FITC	IA6-2	Biolegend
CD19	PerCP-Cy5.5	HIB19	Biolegend
IgG	PE-Cy7	QA19A42	Biolegend
CD38	APC	HIT2	Biolegend
CD27	APC-Cy7	M-T271	Biolegend
IgM	BV421	MHM-88	Biolegend
IgA	BV510	DLDB4	Becton Dickinson
CD45RA	FITC	HI100	Biolegend
CXCR3	PE	CXCR3-173	Biolegend
CD8	PerCP-Cy5.5	SK1	Biolegend
CCR4	PE-Cy7	2G12	Biolegend
CCR6	APC	G034E3	Biolegend
CD4	APC-Cy7	GK1.5	Biolegend
CCR7	BV421	G043H7	Biolegend
CD3	BV510	OKT3	Becton Dickinson
CD56	BV421	MY31	Becton Dickinson
CD25	PE	3C7	Biolegend
CD4	PE-Cy7	SK3	Biolegend
CD127	APC	A7R34	Biolegend

Supplementary table S1. Specificity, clone, fluorochrome and manufacturer of the different monoclonal antibodies used in characterization assays

Marker	Mean pre	Mean post	Median pre	Median post	p_value	FDR
cDC1	0.09	0.19	0.09	0.17	0.04	0.29
cDC1 CCR2^{+}	504.38	888.25	480.50	806.50	0.04	0.29
HLA-DR^{+}	22.55	27.76	23.50	27.40	0.07	0.37
pDC CCR2^{+}	2711.50	3070.63	2671.00	3276.00	0.16	0.48
Non classical monocytes	12.44	9.17	13.25	8.58	0.26	0.48
cDC2 CCR2^{+}	2138.50	2707.88	2259.50	2507.50	0.26	0.48
pDC	1.02	1.41	1.15	1.24	0.26	0.48
HLA-DR^{+} CD11c^{+}	14.70	17.14	13.80	16.70	0.26	0.48
Transitional monocytes	3.84	3.13	3.46	3.31	0.33	0.48
cDC CCR2^{+}	4366.63	5259.25	4300.50	5134.00	0.33	0.48
Classical monocytes CCR2^{+}	5161.25	6212.63	4728.50	5738.50	0.33	0.48
cDC	53.18	55.88	57.95	53.70	0.48	0.65
Non classical monocytes CCR2^{+}	81.08	68.89	74.50	71.95	0.58	0.71
Transitional monocytes CCR2^{+}	2091.00	2400.50	1643.00	2012.00	0.67	0.77
Classical monocytes	71.33	74.08	75.70	72.45	0.78	0.83
cDC2	2.26	2.53	2.31	2.29	1.00	1.00
Plasmablast IgG	19.11	33.95	17.60	31.40	0.01	0.12
IgM memory CD27^{-}	26.91	45.41	25.55	46.80	0.01	0.12
IgG classical memory	16.87	39.62	10.63	43.25	0.05	0.25
IgG	4.93	8.67	5.01	9.23	0.05	0.25
Switch memory	43.23	39.53	42.05	41.95	0.16	0.65
IgD	73.33	60.80	76.05	66.15	0.26	0.65
IgA classical memory	17.43	14.18	17.65	15.50	0.33	0.65
Marginal zone	12.49	14.24	12.60	12.55	0.33	0.65
Naive B-cells	34.55	42.85	36.85	42.30	0.33	0.65
CD27^{-} B cells	82.21	80.73	82.30	82.75	0.40	0.67
Plasmablast IgA	30.11	36.01	31.45	36.20	0.40	0.67
B cells	6.62	7.04	5.75	5.92	0.48	0.69
CD27^{+} B cells	15.28	17.30	15.80	15.25	0.48	0.69
Plasmablast IgD	12.51	11.90	9.63	7.15	0.67	0.84
IgA	4.13	4.82	3.27	3.58	0.67	0.84
Plasmablast	6.31	6.09	5.40	5.41	0.78	0.92
IgM memory	16.61	16.26	18.00	14.75	1.00	1.00
Plasmablast IgM^{+}	19.38	21.01	16.90	19.20	1.00	1.00
IgM	55.73	60.60	56.80	63.05	1.00	1.00
CD4 memory	61.31	54.86	65.00	54.75	0.02	0.50
CD8 TEMRA	18.16	23.58	15.80	19.80	0.05	0.52
CD8 low	4.39	5.70	3.21	4.35	0.07	0.52
Th17	38.14	43.55	33.35	43.70	0.09	0.52
CD4 naive	30.33	37.56	24.85	37.00	0.11	0.52
CD8 high	94.43	93.22	95.45	94.55	0.17	0.55
CD8 memory	17.09	13.99	15.90	9.89	0.20	0.55
Th2	55.17	52.22	58.85	52.55	0.24	0.55
CD4	58.09	56.92	62.45	58.45	0.24	0.55
CD3	52.13	55.41	49.45	53.40	0.28	0.55
CD8 naive	21.39	20.17	18.45	14.85	0.33	0.55
Th1/Th17	33.35	38.30	26.95	37.40	0.33	0.55
CD8 CCR6^{+}	5.66	6.24	3.76	5.78	0.33	0.55
CD4 CCR4^{+}	46.89	42.68	45.50	41.05	0.39	0.59
Th1	56.40	55.51	65.85	55.55	0.44	0.60
CD4 effector memory	7.78	6.85	5.69	1.59	0.58	0.73
CD8 effector memory	43.37	42.26	37.75	38.80	0.72	0.83
CD8	36.35	36.61	33.10	34.40	0.72	0.83
CD4 CXCR3^{+}	11.19	10.43	9.27	8.66	0.88	0.96
CD4 TEMRA	0.57	0.76	0.51	0.49	0.96	0.96
Double CD4 CD8	1.76	1.97	1.50	1.20	0.96	0.96
CD8 CD127^{-}	55.09	30.05	49.00	28.20	0.01	0.09
CD8 CD127^{+}	45.98	64.71	54.05	64.25	0.01	0.09
NKT CD8^{high}	37.98	48.68	36.05	50.20	0.14	0.38
NK CD25^{+}	7.93	0.65	0.74	0.58	0.21	0.42
NKT CD25^{+}	9.29	1.03	4.89	0.45	0.21	0.42
NKT CD8^{-}	45.64	40.59	51.65	39.05	0.31	0.48
Early NK	74.04	76.15	83.85	89.50	0.33	0.48
Tregs	6.41	7.27	5.56	6.71	0.33	0.48
NK	9.97	9.34	7.05	10.10	0.48	0.61
Terminal NK	7.02	6.19	5.75	4.53	0.58	0.61
NKT	15.66	9.45	10.43	9.28	0.58	0.61
NKT CD8^{dim}	12.14	9.47	9.57	11.05	0.58	0.61
Mature NK	12.02	11.57	1.97	3.38	1.00	1.00

Supplementary Table S2. Summary of pre- versus post-vaccination changes in immune subsets among responders. For each population, the table reports the mean and median frequencies before and after vaccination, the paired Wilcoxon test p-value, and the corresponding FDR-adjusted q-value. Responders were defined as patients achieving anti-HBs ≥ 100 IU/L following completion of the Engerix-B® vaccination schedule (n = 8).

Marker	Mean pre	Mean post	Median pre	Median post	p_value	FDR	Marker	Mean pre
pDC CCR2^{+}	2521.50	4288.30	2365.00	4298.50	1766.80	1318.00	0.01	0.12
cDC CCR2^{+}	3794.70	6112.80	2926.00	5562.50	2318.10	2540.00	0.02	0.12
Transitional monocytes CCR2^{+}	1808.90	3386.20	1618.50	2922.50	1577.30	1297.00	0.02	0.12
cDC1c CCR2^{+}	809.30	1457.30	516.00	1175.00	648.00	399.50	0.06	0.24
cDC	56.89	67.74	53.80	65.75	10.85	15.65	0.09	0.25
HLA-DR^{+}	22.02	29.21	24.05	29.95	7.20	5.45	0.14	0.25
HLA-DR^{+} CD11c^{+}	13.37	20.00	14.20	18.65	6.63	4.50	0.14	0.25
Classical monocytes	60.43	74.99	67.45	77.05	14.56	6.65	0.14	0.25
Classical monocytes CCR2^{+}	5600.40	7176.80	3834.50	6425.00	1576.40	1971.00	0.14	0.25
cDC2 CCR2^{+}	2489.30	2838.80	1715.00	2578.00	349.50	555.50	0.17	0.27
Non classical monocytes CCR2+	17.86	10.97	13.05	10.45	-6.90	-2.35	0.24	0.35
Non classical monocytes	85.49	90.01	74.50	84.80	4.52	28.75	0.51	0.68
cDC2	3.52	2.63	2.86	2.96	-0.89	-0.37	0.57	0.71
Transitional monocytes	5.53	4.25	3.37	3.36	-1.27	0.47	0.65	0.74
cDC1c	0.14	0.13	0.13	0.13	0.00	0.02	0.92	0.98
pDC	2.31	1.37	1.14	1.42	-0.93	0.00	1.00	1.00
CD27^{-} B cells	84.78	62.54	86.05	64.55	-22.24	-23.25	0.01	0.03
CD27^{+} B cells	13.10	34.44	12.30	31.90	21.34	22.30	0.01	0.03
IgG classical memory	20.44	44.39	14.35	47.25	23.95	30.75	0.01	0.03
Plasmablast IgG	12.37	29.66	10.90	30.55	17.29	19.78	0.01	0.03
IgM memory	10.43	6.98	10.55	6.69	-3.45	-3.43	0.02	0.07
Switch memory	46.47	37.24	45.65	36.50	-9.23	-9.70	0.02	0.07
IgD	66.28	71.21	69.65	73.55	4.93	3.90	0.04	0.09
Naive B-cells	34.56	50.16	31.30	47.65	15.60	13.20	0.05	0.10
Plasmablast	14.23	8.34	11.07	4.80	-5.89	-4.84	0.07	0.15
IgG	8.77	12.60	2.66	11.30	3.82	6.08	0.11	0.21
Marginal zone	12.61	18.68	10.55	12.55	6.06	6.62	0.14	0.23
IgM memory CD27^{-}	31.15	39.09	24.15	34.95	7.94	10.20	0.20	0.31
IgA classical memory	12.62	10.46	11.30	11.15	-2.16	-4.77	0.24	0.34
IgA	6.87	6.00	2.74	5.26	-0.87	2.40	0.28	0.36
IgM	53.98	58.34	56.60	57.85	4.36	2.65	0.28	0.36
Plasmablast IgA	34.91	42.89	35.85	43.85	7.98	6.10	0.33	0.39
Plasmablast IgM^{+}	12.75	17.00	9.74	14.25	4.24	1.31	0.39	0.43
Plasmablast IgD	7.67	10.07	4.25	9.42	2.40	1.80	0.44	0.47
B cells	7.34	7.12	7.68	7.63	-0.22	0.54	0.96	0.96
CD4 memory	61.31	54.86	65.00	54.75	-6.45	-3.40	0.02	0.50
CD8 TEMRA	18.16	23.58	15.80	19.80	5.42	5.00	0.05	0.52
CD8 low	4.39	5.70	3.21	4.35	1.31	1.19	0.07	0.52
Th17	38.14	43.55	33.35	43.70	5.41	9.00	0.09	0.52
CD4 naive	30.33	37.56	24.85	37.00	7.23	8.60	0.11	0.52
CD8 high	94.43	93.22	95.45	94.55	-1.21	-1.05	0.17	0.55
CD8 memory	17.09	13.99	15.90	9.89	-3.10	-1.40	0.20	0.55
Th2	55.17	52.22	58.85	52.55	-2.95	-7.20	0.24	0.55
CD4	58.09	56.92	62.45	58.45	-1.17	-0.90	0.24	0.55
CD3	52.13	55.41	49.45	53.40	3.28	1.95	0.28	0.55
CD8 naive	21.39	20.17	18.45	14.85	-1.22	-3.10	0.33	0.55
Th1/Th17	33.35	38.30	26.95	37.40	4.95	11.40	0.33	0.55
CD8 CCR6^{+}	5.66	6.24	3.76	5.78	0.58	1.27	0.33	0.55
CD4 CCR4^{+}	46.89	42.68	45.50	41.05	-4.21	-2.20	0.39	0.59
Th1	56.40	55.51	65.85	55.55	-0.89	-7.45	0.44	0.60
CD4 effector memory	7.78	6.85	5.69	1.59	-0.94	-4.78	0.58	0.73
CD8 effector memory	43.37	42.26	37.75	38.80	-1.11	-1.10	0.72	0.83
CD8	36.35	36.61	33.10	34.40	0.26	0.25	0.72	0.83
CD4 CXCR3^{+}	11.19	10.43	9.27	8.66	-0.76	1.51	0.88	0.96
CD4 TEMRA	0.57	0.76	0.51	0.49	0.19	-0.08	0.96	0.96
Double CD4 CD8	1.76	1.97	1.50	1.20	0.21	0.02	0.96	0.96
NK CD25^{+}	16.01	0.41	1.64	0.11	-15.60	-1.19	0.04	0.37
Terminal NK	24.95	5.88	9.21	5.23	-19.07	-0.52	0.11	0.37
NKT CD25^{+}	14.31	3.13	2.32	0.34	-11.18	-2.05	0.11	0.37
Tregs	14.47	7.43	9.71	8.03	-7.04	-2.36	0.14	0.37
Early NK	53.74	88.28	41.40	88.40	34.54	42.40	0.14	0.37
CD8 CD127^{-}	38.49	45.37	35.40	36.90	6.88	6.30	0.14	0.37
NKT	27.81	7.51	5.28	3.64	-20.30	-1.62	0.17	0.38
NK	11.79	14.65	12.50	13.40	2.86	2.50	0.21	0.38
NKT CD8^{high}	28.75	46.77	28.90	37.30	18.02	20.80	0.21	0.38
CD8 CD127^{+}	55.19	49.56	59.70	58.80	-5.63	-9.50	0.26	0.42
NKT CD8^{-}	50.53	30.67	63.30	26.80	-19.87	-31.60	0.31	0.46
NKT CD8^{dim}	16.67	18.25	7.29	8.32	1.58	4.29	0.59	0.68
Mature NK	11.54	3.08	1.45	2.61	-8.46	0.48	0.77	0.82

Supplementary Table S3. Summary of pre- versus post-vaccination changes in immune subsets among non-responders. For each population, the table reports the mean and median frequencies before and after vaccination, the paired Wilcoxon test p-value, and the corresponding FDR-adjusted q-value. Non-responders were defined as patients with anti-HBs < 100 IU/L following completion of the Engerix-B® vaccination schedule (n = 10).

Marker	Mean Δ responder	Median Δ responder	Mean Δ non-responder	Median Δ non-responder	p-value between groups	FDR between groups
pDC CCR2$^+$	359.13	307.00	1766.80	1318.00	0.09	0.57
cDC CCR2$^+$	892.63	942.00	2318.10	2540.00	0.13	0.57
cDC1c	0.11	0.08	0.00	0.02	0.16	0.57
cDC	2.70	3.80	10.85	15.65	0.20	0.57
Classical monocytes	2.75	-0.10	14.56	6.65	0.21	0.57
Transitional monocytes CCR2$^+$	309.50	618.00	1577.30	1297.00	0.25	0.57
Non classical monocytes CCR2+	-12.19	-3.20	4.52	28.75	0.25	0.57
cDC1c CCR2$^+$	383.88	270.00	648.00	399.50	0.33	0.58
Transitional monocytes	-0.71	-1.20	-1.27	0.47	0.33	0.58
pDC	0.38	0.32	-0.93	0.00	0.37	0.60
HLA-DR$^+$ CD11c$^+$	2.44	2.93	6.63	4.50	0.48	0.69
Classical monocytes CCR2$^+$	1051.38	1010.00	1576.40	1971.00	0.53	0.71
cDC2	0.27	-0.23	-0.89	-0.37	0.62	0.77
Non classical monocytes CCR2$^+$	-3.27	-4.66	-6.90	-2.35	0.79	0.86
HLA-DR$^+$	5.21	4.30	7.20	5.45	0.82	0.86
cDC2 CCR2$^+$	569.38	127.50	349.50	555.50	0.86	0.86
CD27$^-$ B cells	-1.49	-2.30	-22.24	-23.25	0.00	0.03
CD27$^+$ B cells	2.02	1.64	21.34	22.30	0.00	0.03
Plasmablast	-0.23	0.58	-5.89	-4.84	0.04	0.20
IgD	-12.53	-3.85	4.93	3.90	0.08	0.30
IgM memory CD27$^-$	18.50	18.45	7.94	10.20	0.13	0.37
Switch memory	-3.70	-2.10	-9.23	-9.70	0.13	0.37
IgM memory	-0.35	-1.21	-3.45	-3.43	0.37	0.79
Marginal zone	1.75	3.26	6.06	6.62	0.42	0.79
IgA	0.69	0.69	-0.87	2.40	0.42	0.79
Plasmablast IgD	-0.61	-1.08	2.40	1.80	0.48	0.79
B cells	0.42	0.28	-0.22	0.54	0.48	0.79
Naive B-cells	8.30	6.15	15.60	13.20	0.53	0.79
Plasmablast IgG	14.84	11.65	17.29	19.78	0.59	0.79
IgG	3.75	4.17	3.82	6.08	0.59	0.79
IgM	4.88	-1.40	4.36	2.65	0.79	0.88
IgA classical memory	-3.25	-3.30	-2.16	-4.77	0.79	0.88
IgG classical memory	22.75	31.93	23.95	30.75	0.79	0.88
Plasmablast IgM$^+$	1.63	3.70	4.24	1.31	0.93	0.93
Plasmablast IgA	5.91	7.79	7.98	6.10	0.93	0.93
CD4 TEMRA	-0.78	-0.62	0.19	-0.08	0.01	0.23
CD8 naive	4.84	5.65	-1.22	-3.10	0.03	0.23
CD4 memory	0.45	0.70	-6.45	-3.40	0.03	0.23
CD8 memory	1.65	1.25	-3.10	-1.40	0.05	0.23
CD8 CCR6$^+$	3.41	3.25	0.58	1.27	0.05	0.23
CD8 TEMRA	0.71	1.60	5.42	5.00	0.11	0.36
CD4	1.78	0.80	-1.17	-0.90	0.14	0.36
Th2	0.05	0.40	-2.95	-7.20	0.15	0.36
CD4 CCR4$^+$	4.88	1.75	-4.21	-2.20	0.16	0.36
CD8 effector memory	-7.18	-7.20	-1.11	-1.10	0.20	0.41
double_cd8_high	4.60	1.85	-3.00	-3.05	0.21	0.41
Th17	4.24	1.90	5.41	9.00	0.25	0.44
CD8 high	5.68	0.25	-1.21	-1.05	0.27	0.44
CD8	-1.14	0.00	0.26	0.25	0.33	0.50
CD3	0.04	-0.35	3.28	1.95	0.42	0.61
CD4 naive	3.01	2.20	7.23	8.60	0.48	0.65
Double CD4 CD8	0.22	0.16	0.21	0.02	0.56	0.68
CD4 CXCR3$^+$	-3.24	-1.12	-0.76	1.51	0.59	0.68
CD4 effector memory	-2.74	-3.38	-0.94	-4.78	0.59	0.68
Th1	-6.43	-5.55	-0.89	-7.45	0.79	0.86
Th1/Th17	10.14	9.24	4.95	11.40	0.86	0.86
CD8 low	0.79	1.01	1.31	1.19	0.86	0.86
CD8 CD127$^-$	-25.04	-23.95	6.88	6.30	0.00	0.03
CD8 CD127$^+$	18.74	15.60	-5.63	-9.50	0.01	0.10
Tregs	0.87	1.68	-7.04	-2.36	0.07	0.36
Early NK	2.11	2.05	34.54	42.40	0.25	0.72
NK	-0.63	-0.49	2.86	2.50	0.25	0.72
NK CD25$^+$	-7.28	-0.27	-15.60	-1.19	0.29	0.72
NKT CD8$^-$	-5.05	-1.55	-19.87	-31.60	0.39	0.72
NKT	-6.21	-0.04	-20.30	-1.62	0.39	0.72
NKT CD8^{dim}	-2.67	-0.91	1.58	4.29	0.44	0.72
Mature NK	-0.45	0.80	-8.46	0.48	0.50	0.72
NKT CD25$^+$	-8.25	-4.03	-11.18	-2.05	0.63	0.72
Terminal NK	-0.82	-0.80	-19.07	-0.52	0.63	0.72
NKT CD8^{high}	10.69	4.15	18.02	20.80	0.85	0.85

Supplementary Table S4. Statistical comparison of vaccine-induced changes (Δ) between responders and non-responders.This table summarizes the between-group statistical analysis of immune subset changes induced by HBV vaccination. For each population, Δ values were computed as post-vaccination minus pre-vaccination frequencies. The table reports median Δ values for responders and non-responders, together with the corresponding between-group p-values and FDR-adjusted q-values (Benjamini–Hochberg correction).
